# Colchicine versus Physical Therapy in Knee Osteoarthritis

**DOI:** 10.3390/life12091297

**Published:** 2022-08-24

**Authors:** George Ovidiu Cioroianu, Alesandra Florescu, Anca Emanuela Mușetescu, Teodor Nicușor Sas, Otilia Constantina Rogoveanu

**Affiliations:** 1 Doctoral School of the University of Medicine and Pharmacy of Craiova, 200349 Craiova, Romania; 2 Department of Physical Medicine and Rehabilitation, University of Medicine and Pharmacy of Craiova, 200349 Craiova, Romania; 3 Department of Rheumatology, University of Medicine and Pharmacy of Craiova, 200349 Craiova, Romania; 4 Department of Radiology and Medical Imaging, University of Medicine and Pharmacy of Craiova, 200349 Craiova, Romania

**Keywords:** knee osteoarthritis, physical therapy, colchicine

## Abstract

Background: The treatment of osteoarthritis remains a major challenge due to the unavailability of a disease-modifying medication and the limitations of current therapeutic perspectives, which mainly target the symptoms, not the disease itself. The purpose of our study is to compare the efficacy of colchicine treatment versus physical therapy. Methods: The study included 62 patients diagnosed with knee osteoarthritis (KOA) according to the American College of Rheumatology (ACR) criteria, hospitalized within the time frame of October 2020–March 2022 in the Department of Rehabilitation Medicine and Physical Therapy of the Emergency Clinical County Hospital of Craiova. The participants were randomly divided into two groups. The observation period was 16 weeks long. The first group (31 patients) received colchicine at a dosage of 1 mg/day together with analgesics (acetaminophen < 2 g/day), while the second group (31 patients) received analgesics (acetaminophen < 2 g/day) together with a 16-week plan of physiotherapy. Results: Group II, in which patients underwent physical therapy, demonstrated a statistically significant decrease in both left (*p* < 0.001) and right (*p* = 0.012) knee VAS and WOMAC (*p* = 0.038) scores at 16 weeks, compared to the group treated with colchicine. Regarding the MSUS examination at 16 weeks, there were no significant changes in the structural abnormalities and no improvement in cartilage aspect or thickness. Higher BMI was associated with higher WOMAC score (*p* = 0.012), but not with higher VAS score (*p* = 0.062). Cholesterol and triglyceride levels were associated with high WOMAC (*p* < 0.001; *p* = 0.021) and high VAS (*p* = 0.023; *p* < 0.001) scores. Conclusions: Our study monitored VAS and WOMAC scores in two groups of patients with KOA, showing that physical therapy is more effective than colchicine in reducing symptoms. We found no statistically significant difference in musculoskeletal ultrasound (MSUS) feature improvement during the 16-week study.

## 1. Introduction

Osteoarthritis (OA) is a non-inflammatory arthropathy characterized by the progressive destruction of the articular cartilage to the point of complete disappearance, followed by the process of bone remodeling with the formation of osteophytes and subchondral sclerosis. OA is a degenerative pathology, but it may be associated with inflammatory changes in the synovial membrane and the articular capsule [1,2].

It mainly affects people over the age of 40, with variations in prevalence due to race and geographic area. Thus, knee OA (KOA) is more frequently encountered in African American women rather than in Caucasian individuals, and hip OA is more frequent in the Chinese population. The rate of incidence is strongly influenced by age and is superior in women, but these differences attenuate over the age of 80 [3,4].

Also, there are several risk factors involved in the pathogenesis of OA. The multifactorial etiologic component may be systematized in elements which define general predisposition such as age, sex, heredity, osteoporosis, hormonal status or diabetes mellitus, and local factors involved in the joint biomechanics [5,6].

The currently available imaging methods play an essential role in establishing the diagnosis of musculoskeletal diseases in general, and osteoarthritis in particular. For this purpose, radiography, ultrasonography, computed tomography, and magnetic resonance imaging can be used. There is a series of advantages and disadvantages for each method [7].

Musculoskeletal ultrasound (MSUS) is a widely available, portable imaging method, with high sensitivity and low cost; it is extremely safe, with no radiation exposure, which allows a real-time, dynamic examination. MSUS allows the detection of pathological changes in tendons, ligaments, menisci, and joints, as well as the presence of tenosynovitis, enthesitis, synovitis, fluid, calcifications, osteophytes, and/or bone erosions. It is also useful in guiding therapeutic maneuvers. The limitations are related to the fact that it is unable to visualize deep joints and structures and the lack of a standardized interpretation methodology [8,9].

Radiography has a low cost, wide availability, short scanning time, and high specificity, but it is irradiating and unable to visualize fluid or synovial proliferation, meniscus lesions, and/or Baker’s cysts in KOA [10,11].

The treatment of osteoarthritis remains a major challenge due to the unavailability of a disease-modifying medication and the limitations of current therapeutic perspectives, which mainly target the symptoms, not the disease itself. A complex, individualized treatment of the patient with KOA should have as therapeutic objectives pain amelioration, preservation of joint function, prevention of structural progression, and implicitly combating disability, with emphasis on the active participation of the patient through understanding this degenerative pathology, life education, and physical activity [12,13]. 

Non-pharmacological therapy includes physical exercises such as stretching, isometric exercises to contract de quadriceps femoris and the gluteus muscles, losing weight, and physical therapy such as electrotherapy, hydro-thermotherapy, tai chi, or yoga. Pharmacological therapy includes symptomatic fast-acting drugs for osteoarthritis (SyFADOA) such as topicals, analgesics, non-steroidal anti-inflammatory drugs (NSAIDs), and intra-articular administration of glucocorticoids or hyaluronic acid, but also platelet-enriched plasma (PRP) and symptomatic slow-acting drugs for osteoarthritis (SySADOA) such as glucosamine, chondroitin, collagen derivates, unsaponifiable soy, and avocado extracts [14,15].

In case of non-pharmacological therapy failure, KOA patients benefit from surgical treatment, which includes arthroscopy, osteotomy, and knee arthroplasty. Knee arthroscopy is considered a conservative therapeutic approach in KOA and has limited indications such as meniscus tears, damaged cruciate ligaments, Baker’s cysts, severe synovial proliferation, and even certain fractures of the knee bones. The indications for osteotomy have decreased over the past few years; however, high tibial osteotomy is still an eligible therapeutic method for young patients with varus deformity and KOA. For severe and advanced KOA, the surgical options range from endoprosthesis to total knee arthroplasty [16]. 

Colchicine, a medication commonly used in gout patients, has been used to treat KOA patients. It is believed to reduce pain and inflammation due to the observation that it might block the inflammasome-mediated pathway. In the case of patients with osteoarthritis without any signs of clinical gout, researchers have found that uric acid levels in the synovial fluid are linked to interleukin (IL)-18, IL-1β, and radiological severity of OA. OA it is thought to aid the local nucleation of monosodium urate (MSU) crystals. Also, the deposition of MSU, which leads to the progression of OA, may be favored by the cartilage degradation in OA. Chondrocyte viability and function are also impaired by MSU deposition, leading to further cartilage damage. This view of the pathophysiology of OA may attest to the use of colchicine in KOA [17,18,19].

The purpose of our study is to compare the efficacy of colchicine treatment versus physical therapy. 

## 2. Materials and Methods

### 2.1. Patients

The study included patients diagnosed with knee osteoarthritis according to the American College of Rheumatology (ACR) criteria [20], hospitalized within the time frame of October 2020–March 2022 in the Department of Rehabilitation Medicine and Physical Therapy of the Emergency Clinical County Hospital of Craiova. The study was approved by the local institutional ethics committee of the University of Medicine and Pharmacy of Craiova (Registration No. 148/11.07.2022), according to European Union Guidelines (Declaration of Helsinki).

The inclusion criteria were age over 18 years, diagnosis of knee osteoarthritis, Kellgren–Lawrence (KL) score ≥ 2 [21], and pain lasting for more than 3 months. The exclusion criteria consisted of the presence of other rheumatic pathologies, recent surgery of the knee, and recent trauma or infection of the knee joint.

### 2.2. Demographic Characteristics and Assessment of Clinical, Laboratory and Imaging Data

All the patients underwent physical examination, blood tests, radiographs, and musculoskeletal ultrasound of the affected knee. 

The clinical assessment included patient history, general clinical examination including the calculation of body mass index (BMI), and also specific evaluation of the knee joint such as inspection of the knee, palpation for points of tenderness, assessment of joint effusion, range-of-motion testing, evaluation of ligaments for injury or laxity, and assessment of the menisci. Patient pain and severity were assessed using the visual analog scale (VAS) of 100 mm. The Western Ontario and McMaster Universities Arthritis Index (WOMAC) was used for evaluating pain with a possible score range of 0–20, stiffness from 0–8, and physical function from 0–68. The questions were scored on the following scale: 0—none, 1—mild, 2—moderate, 3—severe, and 4—extreme [22]. 

The evaluated biological parameters consisted of complete blood count (CBC); liver enzymes; serum creatinine; erythrocyte sedimentation rate (ESR), with a normal range < 10 mm/h; C-reactive protein (CRP), with a normal range < 5 mg/L; cholesterol (COL), with a normal range < 200 mg/dL; and triglycerides (TG), with a normal range < 150 mg/dL.

Ultrasound (US) examinations were performed on an Esaote MyLabX6 US system, using a multi-frequency probe of 6–18 MHz. Grey-scale and power Doppler (PD) techniques in both longitudinal and transverse planes were used in order to evaluate both the inflammatory (joint effusion, synovial proliferation, Baker’s cysts) and structural (osteophytes, femoral hyaline cartilage abnormalities, protrusion of the medial and lateral meniscus) lesions.

Plain radiographs in anteroposterior incidence were used to evaluate the presence of osteophytes, the abnormalities of the joint space, and subchondral sclerosis, graded according to the Kellgren–Lawrence (KL) scale from 0–4 as follows: grade 0—normal, grade 1—doubtful, grade 2—mild, grade 3—moderate, and grade 4—severe. 

### 2.3. Treatment and Outcome

The participants were randomly divided into two groups. The first group received colchicine at a dosage of 1 mg/day together with analgesics (acetaminophen < 2 g/day), while the second group received analgesics (acetaminophen < 2 g/day) together with a 16-week plan of physiotherapy. 

Patients were prescribed interferential current of medium frequency (ICMF), low-level laser therapy (LLLT), and short-wave therapy (SWT).

For the ICMF, we used four poles, applied in an x pattern on the knee joints. The electrodes’ dimensions were 10/10 cm each. The amplitude variation of the medium frequency (AMF) was set to 100 Hz and the spectrum to 0 for 5 min; the AMF was then set to 0 and the spectrum to 0–100 Hz for 10 min. The ICMF was applied once a day for 7 days at weeks 0 and 8.

The LLLT was applied on five points on both knee joints, using an energy flow of 6 J/cm^2^, with a continuous frequency and a daily application for 7 days at weeks 0 and 8. 

The SWT was applied in a capacitor field on both knee joints, using across-application and capacitive applicators. The applied doses were oligo-thermal (20–30 Watts) for 15 min, once per day for 7 days at weeks 0 and 8.

The observation period was 16 weeks long. The patients were re-examined at 8 and 16 weeks. Data regarding VAS and WOMAC were collected on every examination, and MSUS was performed at baseline and week 16. 

### 2.4. Statistical Analysis 

Statistical analysis of the data was performed using GraphPad Prism 9 for Windows. The relationship between the variables was analyzed using the unpaired t-test and the Pearson/Spearman’s coefficient for evaluating correlations. Values of *p* < 0.005 were considered statistically significant. Summary statistics of the mean ± standard deviation (SD) are presented for continuous variables.

## 3. Results

**Figure 1:**
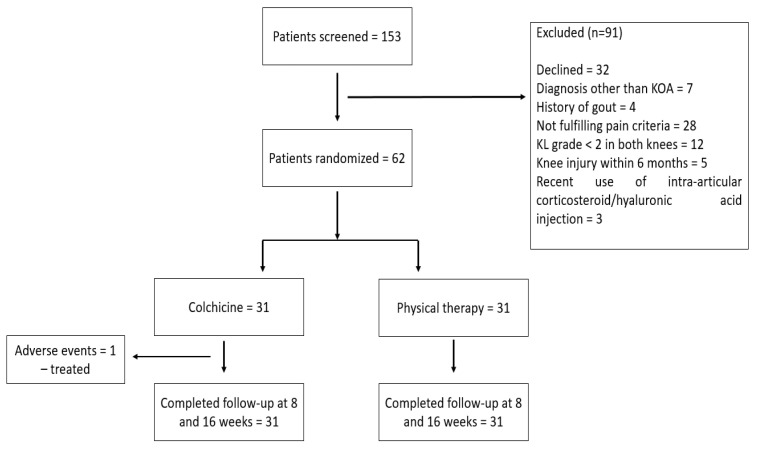
Diagram for patient recruitment and follow-up.

We screened a total of 153 patients with KOA from 2020–2022 and randomized 62 patients (56 females, 6 males) with mean age 65.85 ± 7.42 years. All 62 patients completed the 16-week follow-up (Figure 1). 

Almost all the patients had bilateral KOA, with only 4.83% of patients having undergone knee replacement surgery. Regarding BMI, 17.74% of patients had normal weight, 29.03% of patients were overweight, 24.19% had grade 1 obesity, 22.58% had grade 2 obesity, and 6.46% had grade 3 obesity. Regarding the comorbidities, type 2 diabetes was encountered in 14.51% of patients, while 46.77% had arterial hypertension. 

The descriptive parameters of the patients included in the study are presented in Table 1.

**Figure 2:**
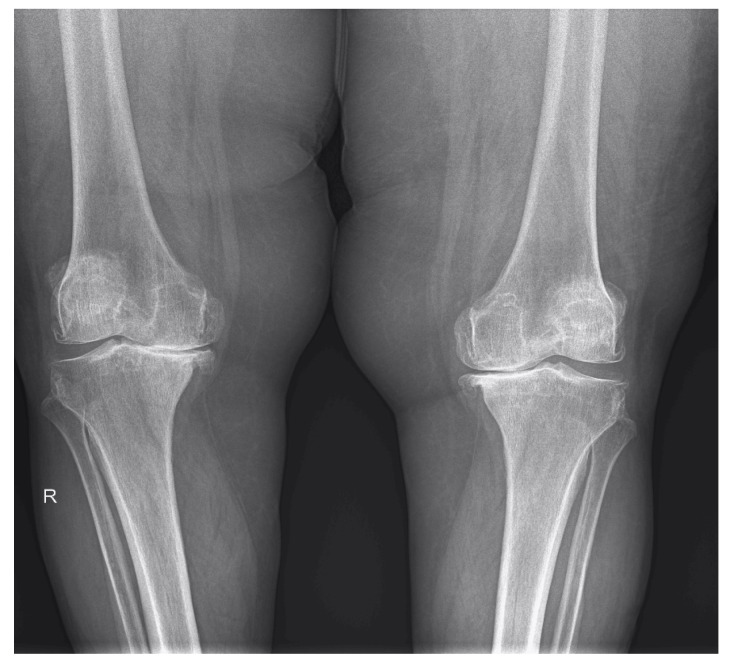
Radiograph of the knees in anterior view showing marked reduction of the joint space, predominantly in the medial compartment, subchondral sclerosis of the tibial plateau, and medial and lateral femoral and tibial osteophytes. R—right.

**Figure 3:**
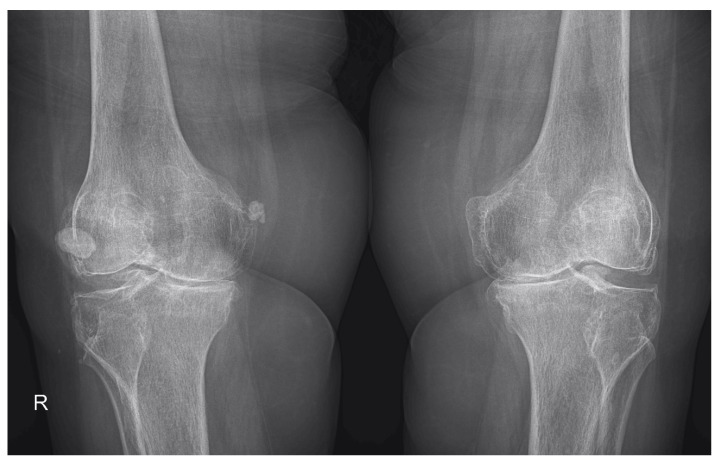
Radiograph of the knees in anterior view showing marked reduction of the joint space, predominantly in the medial compartment, subchondral sclerosis of the tibial plateau, medial and lateral femoral and tibial osteophytes, and ectopic calcifications in the medial aspect of the right femur. R—right.

The MSUS data collected and the abnormalities discovered are presented in Table 2. KL scale 2 was discovered in 32.25% of patients in Group I and 35.48% of patients in Group II, while KL scale 3 was encountered in 46.77% of patients in Group I and 45.16% of patients in Group II. KL scale 4 was found in Group I and II in 20.96% and 16.12% of patients, respectively (Figure 2 and Figure 3).

**Figure 4:**
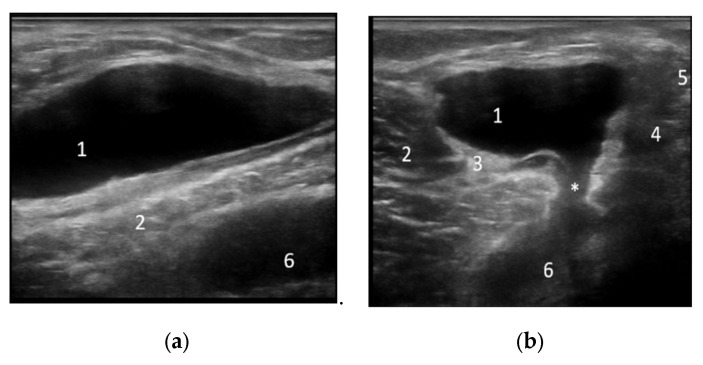
Longitudinal (**a**) and transverse (**b**) scans in grey scale of the posterior knee compartment showing an anechoic collection in the semimembranosus gastrocnemius bursa (Baker’s cyst). 1—collection, 2—medial head of the medial gastrocnemius, 3—the tendon of the medial head of the medial gastrocnemius, 4—semimembranosus tendon, 5—semimembranosus, *—neck, 6—profound section of the Baker cyst.

**Figure 5:**
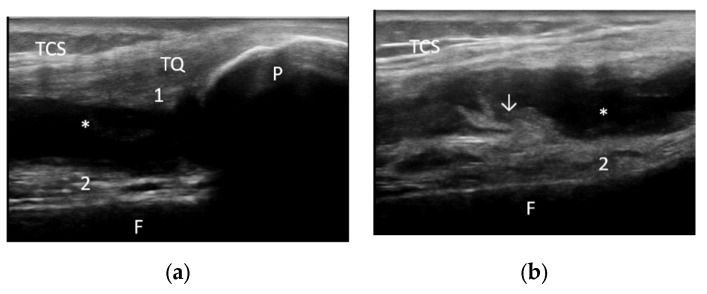
Suprapatellar longitudinal scan (**a**,**b**) in grey scale showing severe joint effusion and grade 2 synovial proliferation. F—femur, P—patella, TQ—quadriceps tendon, 1—suprapatellar fat pad, 2—prefemoral fat pad, *—joint effusion, TCS—subcutaneous cellular tissue, ↓—synovial proliferation.

In Group I, joint effusion was considered mild in 48.38% of the swollen knees, moderate in 45.16%, and severe only in 6.45% of cases. The synovial proliferation was of grade 1 in 11.76% of inflamed joints, grade 2 in 70.58%, and grade 3 in 17.64% of cases. A power Doppler signal was discovered in 35.29% of knees with synovial proliferation. In Group II, joint effusion was considered mild in 47.36% of the swollen knees, moderate in 31.57%, and severe in 21.05% of cases. The synovial proliferation was of grade 1 in 11.76% of inflamed joints, grade 2 in 64.70%, and grade 3 in 23.52% of cases. A power Doppler signal was discovered in 47.05% of knees with synovial proliferation (Figure 4 and Figure 5).

**Figure 6:**
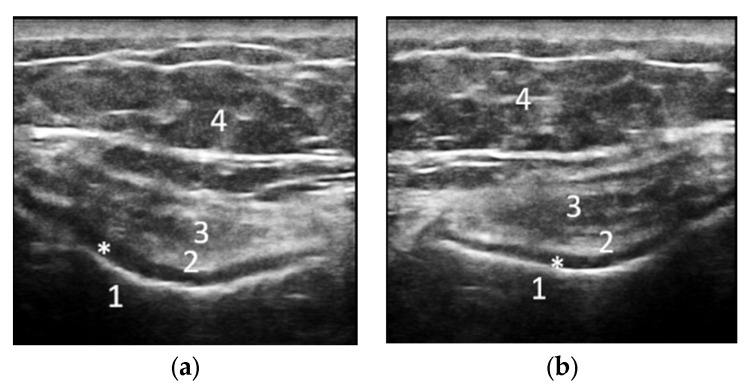
Transverse medial (**a**) and lateral (**b**) scan in grey scale of the femoral trochlea with the knee flexed at 90° showing an hypoechoic, inhomogeneous, reduced in thickness cartilage with irregularities of the osteochondral and chondrosynovial margins. 1—intercondillian femoral trochlea, 2—chondrosynovial interface, 3—quadriceps tendon, *—joint cartilage, 4—subcutaneous cellular tissue.

Cartilage abnormalities were rated as follows (Group I vs. Group II): hypoechoic (93.33% vs. 95.08%), inhomogeneous (100% vs. 100%), reduced in thickness (85% vs. 86.88%), and irregularities of the chondrosynovial and osteochondral margins (55% vs. 44.26%). The thickness of the cartilage was reduced (Group I vs. Group II) between 0–25% in 41.66% vs. 45.90% of cases, 25–50% in 38.33% vs. 32.78% of cases, 50–75% in 16.67% vs. 14.75% of cases, and 75–100% in 3.33% vs. 6.55% of cases (Figure 6). 

### 3.1. Comparison between the Two Groups 

VAS and WOMAC scores were recorded at baseline, 8 and 16 weeks. Mean values ± SD are presented in Table 3. 

We observed a reduction of 6.03% (*p =* 0.214) in mean VAS of the left knee and 7.62% (*p =* 0.268) of the right knee at week 8 compared to baseline in the first group, treated with colchicine. Also, we recorded reductions in mean VAS of the left and right knee of 8.04% (*p =* 0.110) and 13.19% (*p =* 0.051), respectively, at week 16 compared to baseline in Group I. Regarding mean WOMAC, there was a decrease of 1.68% (*p =* 0.729) at week 8 and of 2.88% (*p =* 0.553) at week 16 compared to baseline in Group I. 

In Group II we observed a decrease of 15.49% (*p <* 0.001) at week 8 and of 30.51% (*p* < 0.001) at week 16 in mean VAS of the left knee compared to baseline. Also, we recorded a reduction of 12.84% (*p =* 0.138) at week 8 and of 25.14% (*p =* 0.003) at week 16 in the mean VAS of the right knee compared to baseline in Group II. We noted a decrease of 6.43% (*p =* 0.272) at week 8 and of 12.21% (*p =* 0.037) at week 16 in mean WOMAC compared to baseline in Group II. 

Group II, in which patients underwent physical therapy, demonstrated a statistically significant decrease in both left (*p* < 0.001) and right (*p =* 0.012) knee VAS and WOMAC (*p =* 0.038) scores at 16 weeks, compared to the group treated with colchicine. 

Regarding the MSUS examination at 16 weeks, there were no significant changes in structural abnormalities and no improvement in cartilage aspect or thickness. The inflammatory findings at 16 weeks are depicted in Table 4. 

We observed decreases of 32.26% in overall joint effusion, 58.83% in synovial proliferation, and 50% in overall Baker’s cysts in Group I at 16 weeks (*p =* 0.399). In Group II, we registered decreases of 47.37% in overall joint effusion, 58.83% in synovial proliferation, and 55.56% in overall Baker’s cysts at 16 weeks (*p =* 0.330). 

### 3.2. Associations between Clinical and Paraclinical Data 

Higher BMI was associated with higher WOMAC score (*p =* 0.012) but not with higher VAS (*p =* 0.062). Cholesterol and triglyceride levels were associated with high WOMAC (*p <* 0.001; *p =* 0.021) and high VAS (*p =* 0.023; *p =* 0.001) scores. 

ESR and CRP values were not associated with either VAS (*p =* 0.065; *p =* 0.054) or WOMAC (*p =* 0.078; *p =* 0.056) scores. 

## 4. Discussions

As far as we know, this is the first study comparing colchicine treatment in knee osteoarthritis with physical therapy. However, the limitations of our study include the fact that not many patients were enrolled due to the presence of other pathologies, but also due to the lack of will to participate in a 16-week study during the COVID-19 pandemic. 

The treatment of KOA followed the guidelines stated by the European Alliance of Associations for Rheumatology (EULAR) and European Society for Clinical and Economic Aspects of Osteoporosis, Osteoarthritis and Musculoskeletal Diseases (ESCEO). The ESCEO guidelines were most recently updated in 2019. 

The recommendations state that both non-pharmacological and pharmacological treatment options should be used in combination. A core set of principals advise the information and education of the patients with KOA and emphasize weight loss and regular exercise. Regarding pharmacological treatment, chronic SySADOA should be used to manage symptoms, together with acetaminophen as needed. If the symptoms persist, topical NSAIDs should be used. In case of malalignment, referral to a physical therapist is recommended for knee braces or insoles. Also, walking aids, thermal agents, mechanotherapy or manual therapy, bandage tape, hydrotherapy and aquatic exercises, and tai chi are non-pharmacological therapies that can be added at any time. If the patient is still symptomatic, advanced pharmacological treatment with NSAIDs is recommended. If NSAIDs fail to alleviate symptoms, intra-articular hyaluronate or corticosteroids can be utilized. The last step of the non-pharmacological therapeutic arsenal consists of short-term weak opioids and/or duloxetine. If the quality of life is poor and/or the patient is severely symptomatic, surgical treatment is needed, which consists of either total joint replacement or unicompartmental knee replacement. If surgery is contraindicated, opioid analgesics may be used [23].

Colchicine has been studied in several trials, with the results regarding efficacy in symptom reduction being contrasting. A study conducted by Leung et al., conducted on 109 patients demonstrated a lack of effectiveness in symptom and inflammation reduction in knee osteoarthritis (COLKOA trial), with colchicine failing to meet the primary outcome over a 16-week period compared to a placebo. This conclusion is similar to the one reached in our study. We found that the group treated with physical therapy demonstrated a statistically significant decrease in both left (*p* < 0.001) and right (*p* = 0.012) knee VAS and WOMAC (*p* = 0.038) scores at 16 weeks, compared to the group treated with colchicine [17]. 

A meta-analysis performed by Zhu et al., in 2021 which analyzed six randomized placebo-controlled trials and one non-placebo-controlled trial suggested that even if colchicine is a safe alternative in knee osteoarthritis, no statistically significant differences between colchicine and a placebo were recorded in pain management and function improvement, as demonstrated also in our study [24].

However, two studies conducted by Das et al., in 2002 found that colchicine combined with either nimesulide or intra-articular steroid injections had better outcomes on symptoms than a placebo paired with either of those treatments at 16 and 20 weeks [25,26]. 

A study by Deyle et al., comparing physical therapy with intra-articular glucocorticoid injections on a group of 156 patients with knee osteoarthritis found that the patients who underwent physical therapy had better outcomes regarding pain and functional disability than those who received the intra-articular injections. In our study, physical therapy aided in improving VAS and WOMAC scores, attesting to its efficacy in reducing pain and improving physical function [27]. 

A study conducted by Wang et al., on 204 patients with knee osteoarthritis comparing tai chi with physical therapy found no statistically significant difference between the two groups in WOMAC scores at 12 weeks, with the benefits remaining even at 52 weeks. As in our study, the WOMAC scores were reduced considerably compared to the colchicine group [28]. 

Samuels conducted a study on 80 patients with KOA comparing hyaluronic acid intra-articular injections to physical therapy, which showed that there was no statistically significant difference between the two methods of treatment, both being equally effective in reducing pain and improving quality of life [29]. 

In a study conducted in 2022 on 92 patients with KOA by Tuna et al., the authors evaluated the effect of physical therapy and exercise on pain and functional capacity, showing that physical therapy and exercise had beneficial effects on pain in all patients. However, regarding knee functional capacity, physical therapy was more effective only on patients with KL 1 [30]. 

Also, we observed a reduction in inflammatory changes detected by MSUS in both the colchicine and physical therapy groups, but no improvement in structural changes due to KOA in both groups.

A study by Raud et al., found that KOA is influenced by weight levels. They found a graded relation between obesity stages and clinical consequences in patients with KOA, with the participants with higher BMI having higher pain scores, an association also demonstrated in our study (higher BMI was associated with higher WOMAC scores—*p* = 0.012) [31]. Several studies in the literature have also emphasized the impact weight has on KOA [32,33,34]. 

Also, in our study we found a statistically significant association with high levels of cholesterol and triglycerides, attesting to the implication of metabolic profile in KOA patients. A study conducted by Xie showed that there is a positive association between the prevalence of metabolic syndrome and KOA [35]. 

There is no ideal therapeutic algorithm established in KOA patients, but the studies in the literature attest to the efficacy of weight loss, combined with physical therapy and symptomatic medication. It is possible that in the future, new disease-modifying medication will emerge, but for now physical therapy is considered an effective alternative to medication in patients with KOA [36,37,38].

## 5. Conclusions

Our study monitored VAS and WOMAC scores in two groups of patients with KOA, showing that physical therapy is more effective than colchicine in reducing symptoms. We found no statistically significant difference in MSUS feature improvement during the 16-week study.

## Figures and Tables

**Table 1:** Demographic, clinical, and laboratory data of the study group at baseline.

Demographic, Clinical and Laboratory Features	Patients n = 62	Group I (Colchicine) n = 31	Group II (Physical Therapy) n = 31
Sex (patients, %)	56 females (90.32%); 6 males (9.68%)	28 females (90.32%); 3 males (9.68%)	28 females (90.32%); 3 males (9.68%)
Age (years) (mean, SD)	65.85 (7.42)	65.25 (7.33)	66.45 (7.46)
Disease duration (months) (mean, SD)	29.83 (14.25)	28.36 (13.98)	27.68 (14.20)
Pain at mobilization (patients, %)			
Right	55 (88.70%)	27 (87.09%)	28 (90.32%)
Left	61 (98.38%)	30 (96.77%)	31 (100%)
Crackles (patients, %)			
Right	56 (90.32%)	28 (90.32%)	28 (90.32%)
Left	56 (90.32%)	28 (90.32%)	28 (90.32%)
Patellar tap test (patients, %)			
Right	9 (14.51%)	4 (12.90%)	5 (16.12%)
Left	13 (20.96%)	8 (25.80%)	5 (16.12%)
VAS (mm) (mean, SD)			
Right	61.19 (20.22)	63.54 (18.05)	60.32 (22.06)
Left	66.45 (11.37)	64.19 (12.89)	68.70 (9.06)
WOMAC total	48.19 (10.06)	48.29 (9.11)	48.09 (10.98)
ESR (mm/h) (mean, SD)	29.56 (22.79)	30.51 (22.37)	28.61 (23.16)
CRP (mg/l) (mean, SD)	22.60 (57.54)	23.02 (53.54)	22.17 (61.28)
Cholesterol (mean, SD)	216.11 (41.63)	208.87 (44.14)	223.35 (37.59)
Triglycerides (mean, SD)	116.67 (50.36)	111.96 (49.17)	121.38 (51.10)

**Table 2:** Musculoskeletal ultrasound findings at baseline.

MSUS Findings	Patients		Group I (Colchicine)		Group II (Physical Therapy)	
	Right n (%) n = 62	Left n (%) n = 62	Right n (%) n = 31	Left n (%) n = 31	Right n (%) n = 31	Left n (%) n = 31
*Inflammatory*						
Joint effusion	33 (53.22%)	36 (58.06%)	16 (51.61%)	15 (48.38%)	17 (54.83%)	21 (67.74%)
Synovial proliferation	21 (33.87%)	13 (20.96%)	9 (29.03%)	8 (25.80%)	12 (38.70%)	5 (16.12%)
Baker’s cyst	8 (12.90%)	9 (14.51%)	5 (16.12%)	3 (9.67%)	3 (9.67%)	6 (19.35%)
*Structural*						
Osteophytes	52 (83.87%)	57 (91.93%)	22 (70.96%)	30 (96.77%)	30 (96.77%)	27 (87.09%)
Cartilage	59 (95.16%)	62 (100%)	29 (93.54%)	31 (100%)	30 (96.77%)	31 (100%)
Medial meniscus	37 (59.67%)	38 (61.62%)	18 (58.06%)	20 (64.51%)	19 (61.29%)	18 (58.06%)
Lateral meniscus	15 (24.19%)	15 (24.19%)	7 (22.58%)	8 (25.80%)	8 (25.80%)	7 (22.58%)

**Table 3:** Baseline, week 8, and week 16 VAS and WOMAC scores.

	Baseline		Week 8		Week 16	
	Group I	Group II	Group I	Group II	Group I	Group II
VAS left	64.19 (12.89)	68.70 (9.06)	60.32 (10.92) *p* = 0.214	58.06 (7.79) *p <* 0.001	59.03 (11.73) *p* = 0.110	47.74 (8.31) *p* < 0.001
VAS right	63.54 (18.05)	60.32 (22.06)	58.70 (15.39) *p* = 0.268	52.58 (17.59) *p* = 0.138	55.16 (14.33) *p* = 0.051	45.16 (15.63) *p* = 0.003
WOMAC	48.29 (9.11)	48.09 (10.93)	47.48 (8.88) *p* = 0.729	45 (10.72) *p* = 0.272	46.90 (8.90) *p* = 0.533	42.22 (10.37) *p* = 0.037

**Table 4:** Ultrasound features at baseline and 16 weeks in the two groups.

MSUS Findings	Group I Baseline		Group II Baseline		Group I 16 Weeks		Group II 16 Weeks	
	Right n (%) n = 31	Left n (%) n = 31	Right n (%) n = 31	Right n (%) n = 31	Right n (%) n = 31	Left n (%) n = 31	Right n (%) n = 31	Left n (%) n = 31
*Inflammatory*								
Joint effusion	16 (51.61%)	15 (48.38%)	17 (54.83%)	21 (67.74%)	10 (32.25%)	11 (35.48%)	9 (29.03%)	11 (35.48%)
Synovial proliferation	9 (29.03%)	8 (25.80%)	12 (38.70%)	15 (16.12%)	4 (12.90%)	3 (9.67%)	6 (19.35%)	3 (9.67%)
Baker’s cyst	5 (16.12%)	3 (9.67%)	3 (9.67%)	6 (19.35%)	2 (6.67%)	2 (6.67%)	1 (3.22%)	3 (9.67%)
